# Multi-Level Transcriptomic and Physiological Responses of *Aconitum kusnezoffii* to Different Light Intensities Reveal a Moderate-Light Adaptation Strategy

**DOI:** 10.3390/genes16080898

**Published:** 2025-07-28

**Authors:** Kefan Cao, Yingtong Mu, Xiaoming Zhang

**Affiliations:** College of Grassland Science/Key Laboratory of Grassland Resources of Ministry of Education, Inner Mongolia Agricultural University, Hohhot 010018, China; caokefan1003@163.com (K.C.); myt100862@outlook.com (Y.M.)

**Keywords:** *Aconitum kusnezoffii*, light intensity, transcriptome, antioxidant enzymes, photomorphogenesis, hormone signaling

## Abstract

Objectives: Light intensity is a critical environmental factor regulating plant growth, development, and stress adaptation. However, the physiological and molecular mechanisms underlying light responses in *Aconitum kusnezoffii*, a valuable alpine medicinal plant, remain poorly understood. This study aimed to elucidate the adaptive strategies of *A. kusnezoffii* under different light intensities through integrated physiological and transcriptomic analyses. Methods: Two-year-old *A. kusnezoffii* plants were exposed to three controlled light regimes (790, 620, and 450 lx). Leaf anatomical traits were assessed via histological sectioning and microscopic imaging. Antioxidant enzyme activities (CAT, POD, and SOD), membrane lipid peroxidation (MDA content), osmoregulatory substances, and carbon metabolites were quantified using standard biochemical assays. Transcriptomic profiling was conducted using Illumina RNA-seq, with differentially expressed genes (DEGs) identified through DESeq2 and functionally annotated via GO and KEGG enrichment analyses. Results: Moderate light (620 lx) promoted optimal leaf structure by enhancing palisade tissue development and epidermal thickening, while reducing membrane lipid peroxidation. Antioxidant defense capacity was elevated through higher CAT, POD, and SOD activities, alongside increased accumulation of soluble proteins, sugars, and starch. Transcriptomic analysis revealed DEGs enriched in photosynthesis, monoterpenoid biosynthesis, hormone signaling, and glutathione metabolism pathways. Key positive regulators (*PHY* and *HY5*) were upregulated, whereas negative regulators (*COP1* and *PIFs*) were suppressed, collectively facilitating chloroplast development and photomorphogenesis. Trend analysis indicated a “down–up” gene expression pattern, with early suppression of stress-responsive genes followed by activation of photosynthetic and metabolic processes. Conclusions: *A. kusnezoffii* employs a coordinated, multi-level adaptation strategy under moderate light (620 lx), integrating leaf structural optimization, enhanced antioxidant defense, and dynamic transcriptomic reprogramming to maintain energy balance, redox homeostasis, and photomorphogenic flexibility. These findings provide a theoretical foundation for optimizing artificial cultivation and light management of alpine medicinal plants.

## 1. Introduction

*Aconitum kusnezoffii* is a perennial herbaceous species in the family Ranunculaceae with high medicinal and economic value [1,2]. It is primarily distributed across northeastern, northwestern, and northern regions of China, where it serves as a key traditional Chinese medicinal herb known for its functions in dispelling wind, removing dampness, warming meridians, and alleviating pain. It is widely used in the treatment of ailments, such as rheumatic arthralgia and palpitations with shortness of breath. The major bioactive constituents of *A. kusnezoffii* are diterpenoid alkaloids, and the aboveground tissues also exhibit considerable pharmacological potential [3,4]. With the progressive depletion of wild resources, artificial cultivation and efficient breeding of *A. kusnezoffii* have emerged as critical areas of research [5]. However, the physiological adaptation mechanisms of this species to environmental factors—particularly its responses to varying light intensities—remain poorly understood.

Light is one of the most essential environmental factors influencing plant growth and development, directly affecting photosynthetic efficiency, energy accumulation, and metabolic regulation [6]. Numerous studies have demonstrated that changes in light intensity can significantly impact plant morphogenesis, chlorophyll biosynthesis, hormonal homeostasis, and reactive oxygen species (ROS) metabolism [7,8]. In medicinal plants such as *Perilla frutescens* [9], *Sarcandra glabra* [10], and *Scutellaria baicalensis* [11], light intensity has been shown to modulate the biosynthesis of secondary metabolites, antioxidant activity, and the accumulation of pharmacologically active compounds. Insufficient light typically leads to reduced chlorophyll content and elevated malondialdehyde (MDA) levels, triggering oxidative stress. In response, plants often activate antioxidant enzymes, such as superoxide dismutase (SOD), peroxidase (POD), and catalase (CAT), to mitigate cellular damage and maintain redox homeostasis.

At the molecular level, light signaling pathways involve key photoreceptors—such as *PHYA/B*—as well as regulatory components like *HY5*, *COP1*, and *PIFs*, which collectively mediate photomorphogenic development and stress responses. In recent years, RNA sequencing (RNA-seq) has been widely employed to investigate plant responses to abiotic stresses, including light, drought, salinity, and temperature, thereby advancing our understanding of stress adaptation mechanisms in non-model species. For instance, transcriptomic analyses in *Salvia miltiorrhiza* [12] and *Verbena officinalis* [13] have revealed how light modulates the expression of genes associated with photosynthesis, carbon metabolism, and antioxidative defense. Nevertheless, a comprehensive investigation into the physiological and transcriptional responses of *A. kusnezoffii* to different light intensities remains lacking, particularly with respect to key regulatory factors involved in light perception, oxidative defense, and metabolic reprogramming.

In this study, *A. kusnezoffii* was subjected to three light intensity treatments—high, moderate, and low—to systematically evaluate the changes in leaf chlorophyll content, soluble sugar, soluble protein, MDA levels, and antioxidant enzyme activities. Additionally, transcriptome sequencing was employed to identify differentially expressed genes (DEGs) and to elucidate key regulatory pathways and response mechanisms. The objective was to uncover the physiological and molecular basis underlying light-regulated responses in *A. kusnezoffii*, thereby providing theoretical support for and molecular insights into its cultivation optimization and medicinal utilization.

## 2. Materials and Methods

### 2.1. Plant Materials and Experimental Design

Two-year-old healthy plants of *A. kusnezoffii* were used as experimental materials. Wild individuals were originally collected from natural habitats in Xing’an League, Inner Mongolia, China. After acclimation, the plants were transplanted and cultivated in the greenhouse of the Medicinal Plant Research Base at Inner Mongolia Agricultural University. Once growth stabilized, morphologically uniform and vigorous individuals were selected for light intensity treatments.

Three light intensity levels were established, high light (A, 790 lx), moderate light (B, 620 lx), and low light (C, 450 lx), controlled using LED plant growth lamps combined with a shading cloth. Each treatment included three biological replicates and lasted for 14 days. Other environmental conditions were kept consistent (temperature of 25 ± 2 °C, relative humidity of 60–70%, and photoperiod of 14 h/10 h). At the end of the treatment, functional leaves were sampled at the same time of day, immediately frozen in liquid nitrogen, and stored at –80 °C for subsequent analyses.

### 2.2. Leaf Anatomical Structure Observation and Measurement

To evaluate the effects of different light intensities on the leaf anatomical structure, functional leaves from plants under 790 lx, 620 lx, and 450 lx treatments were collected for histological sectioning and microscopic observation on the 35th day post-treatment (sampling time: 9:00–10:00 a.m.).

Fresh leaf segments (approx. 5 mm × 5 mm) were fixed in an FAA solution (70% ethanol:formalin:glacial acetic acid = 90:5:5, *v*/*v*/*v*) for 48 h. The samples were dehydrated through an ethanol gradient, cleared, embedded in paraffin, and sectioned (8 μm thickness). Double staining with safranin and fast green was performed.

Sections were observed and imaged using a light microscope (e.g., Olympus BX53, Olympus Corporation, Tokyo, Japan), and anatomical parameters were measured using ImageJ software (version 1.54, National Institutes of Health, Bethesda, MD, USA). Parameters included upper epidermis thickness, lower epidermis thickness, palisade tissue thickness, spongy tissue thickness, total leaf thickness, and palisade/spongy tissue ratio.

For each treatment, 10 visual fields were measured. Data are expressed as means ± standard deviations (SDs) and were subjected to a statistical significance analysis.

### 2.3. Physiological and Biochemical Index Measurements

To investigate the physiological and biochemical responses of *A. kusnezoffii* leaves under different light intensities, the following parameters were determined with three biological replicates per treatment:

(1) Malondialdehyde (MDA) Content

Leaf samples (0.2 g) were analyzed using the thiobarbituric acid (TBA) method. Absorbance was measured at 532 nm and 600 nm, and MDA concentration was calculated based on the difference, reflecting membrane lipid peroxidation.

(2) Catalase (CAT) Activity

CAT activity was determined using UV spectrophotometry at 240 nm by monitoring the decomposition rate of H_2_O_2_.

(3) Peroxidase (POD) Activity

The guaiacol colorimetric method was used. Absorbance was measured at 470 nm to calculate POD activity based on the oxidation rate of guaiacol.

(4) Superoxide Dismutase (SOD) Activity

SOD activity was assessed using the nitro blue tetrazolium (NBT) photoreduction method, measuring inhibition at 560 nm to estimate the plant’s ability to scavenge superoxide anions.

(5) Free Proline (Pro) Content

Samples (0.1 g) were analyzed via the acid ninhydrin method. Absorbance at 520 nm was used to calculate proline content from a standard curve, reflecting the osmotic adjustment capacity.

(6) Soluble Protein (SP) Content

Protein content was determined using the Coomassie Brilliant Blue G-250 method, measuring absorbance at 595 nm and calculating concentrations via a standard curve.

(7) Soluble Sugar (SS) Content

Samples (0.1 g) were assayed using the anthrone method. Absorbance at 620 nm was recorded, and sugar content was estimated based on a glucose standard curve.

(8) Starch Content

Samples (0.03 g) were enzymatically hydrolyzed and analyzed using the anthrone method. Absorbance at 620 nm was measured, and starch content was calculated via a standard curve.

### 2.4. RNA Extraction and Transcriptome Sequencing

#### 2.4.1. RNA Isolation and Library Construction

Total RNA was extracted from *A. kusnezoffii* leaves using the TRIzol reagent. RNA quality and concentration were assessed using a NanoDrop 2000(Thermo Fisher Scientific, Waltham, MA, USA), Qubit Fluorometer(Thermo Fisher Scientific, Waltham, MA, USA), and Agilent 2100 Bioanalyzer(Agilent Technologies, Santa Clara, CA, USA). Samples with an RNA integrity number (RIN) ≥ 7.0 were selected for library construction. Libraries were prepared using the NEBNext Ultra RNA Library Prep Kit (New England Biolabs, Ipswich, MA, USA) and sequenced on the Illumina platform with paired-end 150 bp reads (PE150).

#### 2.4.2. Transcriptome Data Processing

Raw reads were quality-filtered and adapter-trimmed using fastp to obtain clean reads. Transcriptome assembly was performed using Trinity, and annotation was conducted against NR, SwissProt, GO, and KEGG databases. Differentially expressed genes (DEGs) were identified using DESeq2 with a threshold of |log_2_FC| ≥ 1 and *p* < 0.05. GO enrichment and KEGG pathway analyses were performed for a functional categorization of DEGs.

#### 2.4.3. qRT-PCR Validation

To validate the reliability of RNA-seq results, ten representative DEGs related to light regulation were selected for qRT-PCR analysis, including *LHCB17*, *LHCB1*, *ATPβ*, *PetA*, *PFK*, *NCED*, *PP2C*, *GA2ox*, *DELLA*, and *AMY*, which are involved in photosynthesis, carbon metabolism, and hormone signaling pathways.

Primers were designed using Primer 5.0 based on unigene sequences from the *A. kusnezoffii* transcriptome. The *Actin* gene was used as the internal control. Total RNA was extracted as described above, and cDNA was synthesized using the PrimeScript RT reagent Kit (Takara Bio Inc., Shiga, Japan) in a 20 μL reaction system. qRT-PCR was performed using the ABI 7500 Real-Time PCR System with SYBR Premix Ex Taq II (Takara). The cycling conditions were as follows: 95 °C for 30 s, followed by 40 cycles of 95 °C for 5 s and 60 °C for 34 s.

Each reaction was conducted with three technical and three biological replicates. Relative expression levels were calculated using the 2^−ΔΔCt^ method and compared with RNA-seq data (Table 1).

### 2.5. Data Analysis

All physiological and qRT-PCR data were obtained from three biological replicates and are expressed as means ± standard deviations (SDs). A statistical analysis was performed using R software (version 4.2.0). One-way analysis of variance (ANOVA) was conducted, followed by Duncan’s multiple range test for significance comparisons. A *p*-value < 0.05 was considered statistically significant.

Graphical visualization was performed using the ggplot2 package. Differences between treatments were illustrated as bar charts with annotated significance letters. Transcriptome quality control was performed using fastp, and transcript assembly was carried out via Trinity. Functional annotation referred to NR, SwissProt, GO, and KEGG databases. DEGs were screened using DESeq2 (|log_2_FoldChange| ≥ 1, *p* < 0.05). GO classification and KEGG enrichment analysis were performed using the ClusterProfiler package(version 4.10.0) and visualized as bar plots and bubble plots.

## 3. Results

### 3.1. Effects of Light Intensity on Leaf Anatomical Structure in A. kusnezoffii

**Figure 1:**
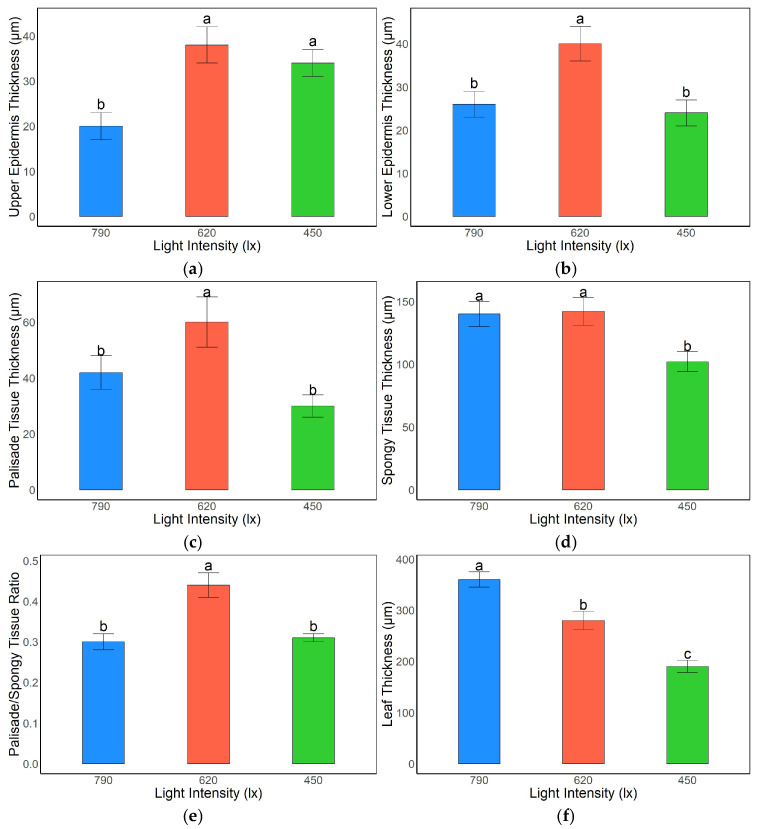
Effects of light intensity on leaf anatomical structure in *A. kusnezoffii*: (**a**) upper epidermis thickness; (**b**) lower epidermis thickness; (**c**) palisade tissue thickness; (**d**) spongy tissue thickness; (**e**) palisade/spongy tissue ratio; (**f**) total leaf thickness. Different lowercase letters indicate significant differences between treatments at *p* < 0.05.

Light intensity exerted significant effects on the anatomical structure of *A. kusnezoffii* leaves. For the upper epidermis thickness, the 620 lx and 450 lx treatments exhibited values of 36.9 μm and 34.2 μm, respectively, both of which were significantly higher than the 20.5 μm observed under 790 lx (*p* < 0.05), indicating that moderate and low light conditions were favorable for upper epidermal cell thickening (Figure 1a). Regarding the lower epidermis, the 620 lx group showed the highest thickness (39.4 μm), which was significantly greater than that of the 790 lx (26.5 μm) and 450 lx (24.3 μm) groups (*p* < 0.05), suggesting that moderate light facilitated the coordinated development of epidermal tissues (Figure 1b).

In terms of mesophyll structure, palisade tissue thickness reached 60.2 μm under the 620 lx treatment, significantly higher than those under 790 lx (43.1 μm) and 450 lx (32.0 μm) conditions (*p* < 0.05), indicating that moderate light intensity promoted the development of photosynthetic tissues (Figure 1c). Spongy tissue thickness was also significantly greater in the 790 lx (139.8 μm) and 620 lx (140.1 μm) groups compared to the 450 lx group (104.5 μm) (*p* < 0.05), reflecting an enhanced development of gas-exchange structures under relatively higher light intensities (Figure 1d).

The palisade-to-spongy tissue thickness ratio under 620 lx was 0.43, which was significantly higher than the ratios observed under 790 lx and 450 lx (both 0.31; *p* < 0.05), suggesting a more favorable mesophyll tissue distribution under moderate light, potentially conducive to higher photosynthetic efficiency (Figure 1e). Notably, total leaf thickness was greatest under 790 lx (353.2 μm), significantly exceeding that of the 620 lx (280.4 μm) and 450 lx (195.3 μm) treatments (*p* < 0.05), implying that strong light may induce overall leaf thickening as an adaptive response to high light stress (Figure 1f).

In summary, *A. kusnezoffii* exhibited the most coordinated epidermal and mesophyll structural development under 620 lx, forming an anatomically optimized leaf architecture suitable for moderate light environments. In contrast, structural imbalances were observed under both low (450 lx) and high (790 lx) light conditions, likely due to limitations or stress imposed by insufficient or excessive light, respectively.

### 3.2. Physiological Responses of A. kusnezoffii to Different Light Intensities

#### 3.2.1. Effects of Light Intensity on MDA Content in *A. kusnezoffii* Leaves

Significant differences in malondialdehyde (MDA) content were observed in *A. kusnezoffii* leaves under different light intensities. The highest MDA content was detected under the 450 lx treatment, reaching 3.65 μmol/g, which was significantly higher than that under 790 lx and 620 lx (*p* < 0.05), indicating that low light conditions aggravated membrane lipid peroxidation. Under 790 lx, the MDA content was 2.57 μmol/g, representing an intermediate level, whereas the lowest value was recorded at 620 lx (1.96 μmol/g), which was significantly lower than in the other two treatments (*p* < 0.05).

**Figure 2:**
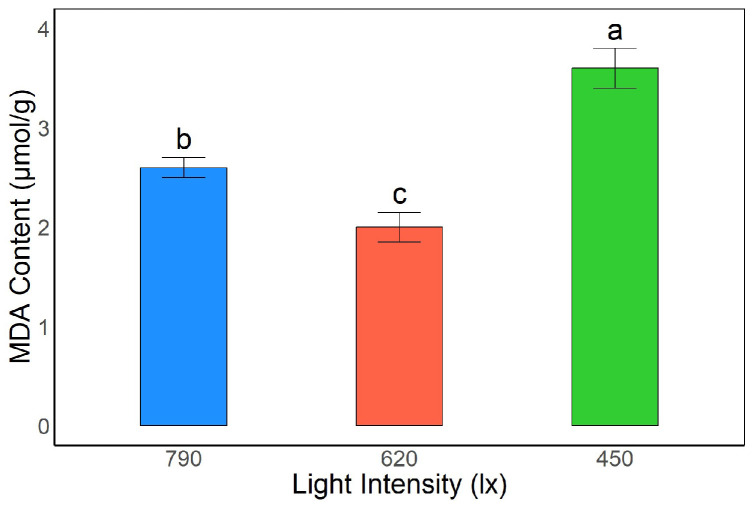
Effects of different light intensities on malondialdehyde (MDA) content in *A. kusnezoffii* leaves. Different lowercase letters indicate significant differences between treatments at *p* < 0.05.

These results suggest that moderate light intensity (620 lx) helps reduce membrane lipid peroxidation, stabilizing membrane systems and alleviating oxidative stress. In contrast, low light conditions (450 lx) led to more severe membrane damage, which may be detrimental to the normal growth and development of *A. kusnezoffii* (Figure 2).

#### 3.2.2. Effects of Light Intensity on Antioxidant Enzyme Activities in *A. kusnezoffii* Leaves

**Figure 3:**
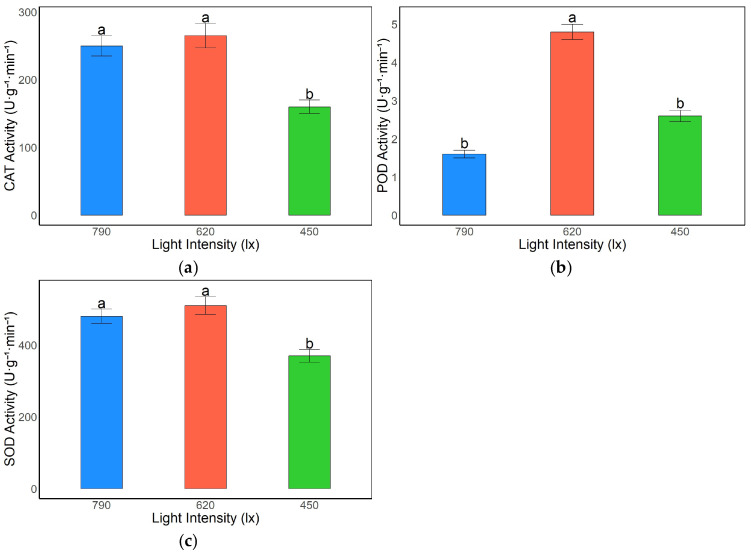
Effects of different light intensities on antioxidant enzyme activities in *A. kusnezoffii* leaves: (**a**) catalase (CAT); (**b**) peroxidase (POD); (**c**) superoxide dismutase (SOD). Different lowercase letters indicate significant differences between treatments at *p* < 0.05.

Light intensity significantly affected the activities of antioxidant enzymes in the leaves of *A. kusnezoffii*. Catalase (CAT) activity was highest under the 620 lx treatment, reaching 267.85 U·g^−1^·min^−1^. This value was slightly higher than that under 790 lx (245.65 U·g^−1^·min^−1^), with no significant difference between them; however, both were significantly higher than the value recorded under 450 lx (165.94 U·g^−1^·min^−1^) (*p* < 0.05). These results indicate that moderate and high light conditions are favorable for the induction of CAT activity, which plays an essential role in alleviating oxidative stress (Figure 3a).

Similarly, peroxidase (POD) activity peaked under 620 lx (4.78 U·g^−1^·min^−1^), significantly exceeding those measured under the 790 lx (1.62 U·g^−1^·min^−1^) and 450 lx (2.59 U·g^−1^·min^−1^) treatments (*p* < 0.05), suggesting that moderate light intensity effectively activates POD activity and enhances the antioxidant capacity of the plant (Figure 3b).

Superoxide dismutase (SOD) activity showed comparable levels under 620 lx and 790 lx, at 510.26 U·g^−1^·min^−1^ and 490.42 U·g^−1^·min^−1^, respectively, with no significant difference between them. Both values were significantly higher than that under 450 lx (382.67 U·g^−1^·min^−1^) (*p* < 0.05), indicating a stronger SOD-mediated response under moderate and high light conditions compared to low light (Figure 3c).

In summary, moderate light intensity (620 lx) was most conducive to the activation of the antioxidant enzyme system in *A. kusnezoffii*, promoting the scavenging of reactive oxygen species (ROS) generated under light stress and thereby enhancing stress tolerance. In contrast, low light conditions (450 lx) resulted in generally lower enzyme activities, potentially impairing the plant’s antioxidative defense capacity.

#### 3.2.3. Effects of Light Intensity on Osmoregulatory Substances and Metabolites in *A. kusnezoffii*

**Figure 4:**
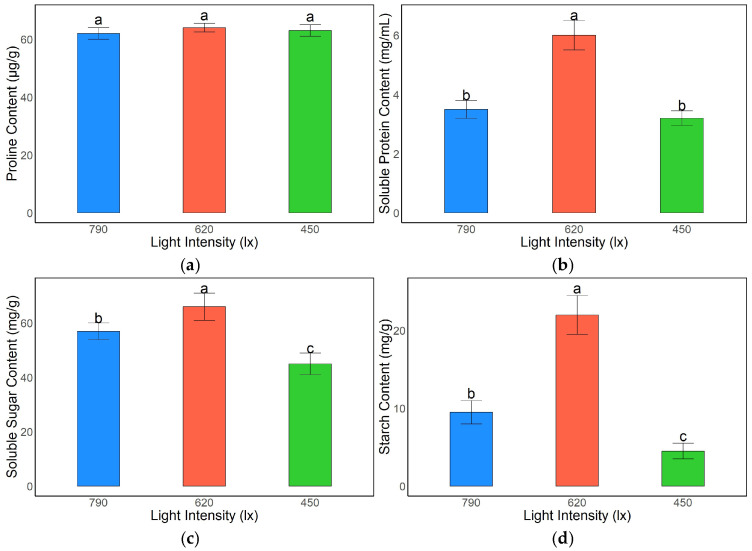
Effects of different light intensities on the contents of osmoregulatory substances and metabolic products in *A. kusnezoffii* leaves: (**a**) proline content; (**b**) soluble protein content; (**c**) soluble sugar content; (**d**) starch content. Different lowercase letters indicate significant differences between treatments at *p* < 0.05.

Light intensity had a pronounced effect on the accumulation of osmoregulatory substances and primary metabolites in *A. kusnezoffii* leaves. Proline content remained relatively stable across the treatments, with values of 61.3, 64.5, and 62.8 μg/g under 790 lx, 620 lx, and 450 lx, respectively, and no significant differences were observed among the groups (*p* > 0.05), suggesting that proline levels were maintained under varying light conditions (Figure 4a).

Soluble protein content was highest under the 620 lx treatment (5.9 mg/mL), which was significantly greater than the levels observed under 790 lx (3.9 mg/mL) and 450 lx (3.7 mg/mL) (*p* < 0.05), indicating that moderate light intensity promoted protein synthesis and accumulation (Figure 4b).

Similarly, soluble sugar content peaked under 620 lx (66.0 mg/g), significantly exceeding that under 790 lx (56.4 mg/g) and 450 lx (44.2 mg/g) (*p* < 0.05). This suggests that appropriate light intensity facilitates carbohydrate accumulation, which may enhance the osmoregulatory capacity (Figure 4c).

Starch content showed the most marked variation among the treatments. The highest value was recorded under 620 lx (21.8 mg/g), which was significantly greater than that under 790 lx (9.5 mg/g) and 450 lx (3.6 mg/g) (*p* < 0.05), indicating that moderate light intensity strongly promoted starch biosynthesis and storage, whereas low light conditions suppressed starch accumulation (Figure 4d).

Collectively, these findings demonstrate that a light intensity of 620 lx is most favorable for the accumulation of osmoregulatory substances and primary metabolites in *A. kusnezoffii*. This may contribute to enhanced physiological adaptability under fluctuating light conditions by maintaining osmotic homeostasis and metabolic stability.

### 3.3. Transcriptome Analysis

#### 3.3.1. Quality Assessment of Sequencing Data

In this study, high-throughput transcriptome sequencing was performed on nine samples of *A. kusnezoffii* leaves under different light intensity treatments. The experimental design included three treatment groups, Group C (control, 450 lx), Group B (moderate light, 620 lx), and Group A (high light, 790 lx), with three biological replicates per group (designated as A1–A3, B1–B3, and C1–C3). Basic sequencing statistics are summarized in Table 2.

The number of clean reads ranged from 20,223,069 to 22,691,284 across all samples, yielding between 6.06 and 6.80 Gb of clean bases, indicating that the sequencing depth was sufficient to support reliable transcriptome assembly and expression analysis. The GC content of the samples ranged from 45.53% to 46.04%, which is consistent with typical plant transcriptomes and shows no apparent bias. Regarding sequence quality, all samples achieved Q30 values above 94%, with the highest values observed in samples B2 and C2 (95.49%). The remaining samples also maintained high Q30 scores, around 95%, demonstrating the high accuracy and stability of the sequencing data (Table 2).

#### 3.3.2. Differentially Expressed Gene (DEG) Analysis

**Figure 5:**
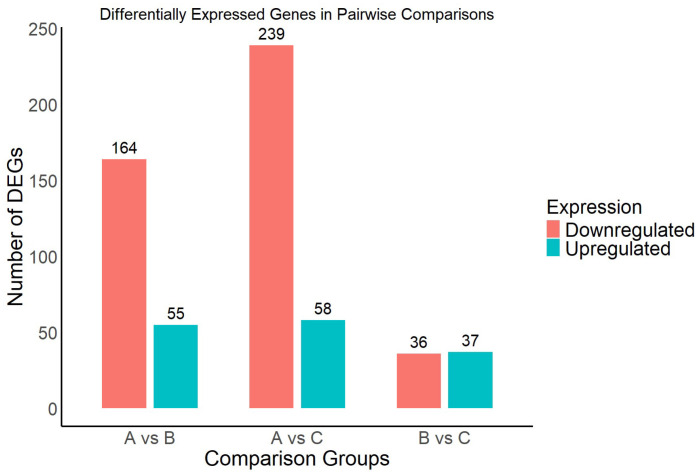
Number of differentially expressed genes (DEGs) in each pairwise comparison group under different light intensities.

To further investigate the molecular mechanisms by which *A. kusnezoffii* responds to varying light intensities, pairwise comparisons were conducted among the three treatment groups to identify differentially expressed genes (DEGs). The results are presented in Figure 5.

A total of 219 DEGs were identified in the comparison between high light (Group A) and moderate light (Group B), including 55 upregulated and 164 downregulated genes. In the high light (A) versus low light (C) comparison, 297 DEGs were detected, with 58 genes upregulated and 239 downregulated. In contrast, the comparison between moderate light (B) and low light (C) yielded 73 DEGs, including 37 upregulated and 36 downregulated genes. Overall, the A vs. C group exhibited the highest number of DEGs, indicating that the transcriptional response of *A. kusnezoffii* was most pronounced under extreme differences in light intensity. This suggests that large shifts in light availability more strongly trigger molecular responses in this species.

Moreover, in all comparison groups, the number of downregulated genes exceeded that of upregulated genes, with the disparity most prominent in the A vs. C comparison. This trend implies that high light conditions may induce widespread repression of specific functional genes. These DEGs provide foundational information for subsequent functional annotation and pathway enrichment analyses and will contribute to elucidating the key regulatory networks underlying light-mediated growth and development in *A. kusnezoffii*.

#### 3.3.3. GO Functional Annotation of Differentially Expressed Genes

**Figure 6:**
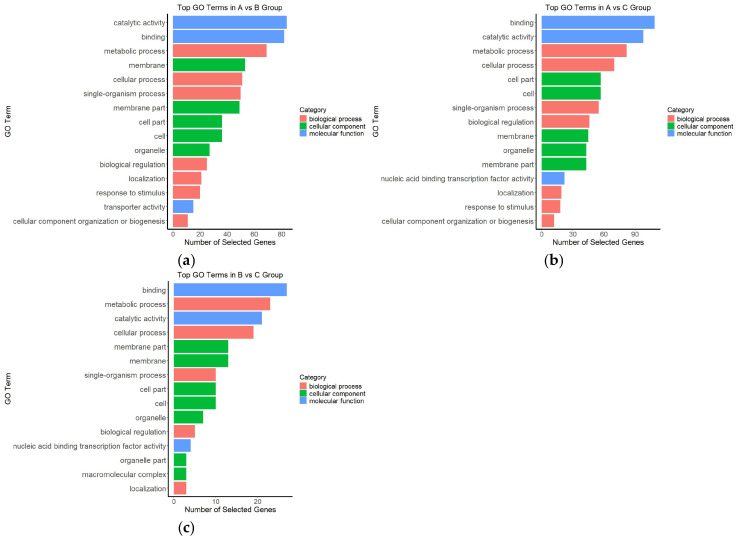
Gene Ontology (GO) enrichment classification of differentially expressed genes under different light intensity comparisons; (**a**) A vs. B; (**b**) A vs. C; (**c**) B vs. C.

To further elucidate the functional changes in gene expression of *A. kusnezoffii* under different light intensities, Gene Ontology (GO) enrichment analysis was performed on the identified differentially expressed genes (DEGs). GO terms were categorized into three main domains: biological process (BP), cellular component (CC), and molecular function (MF). Figure 6 displays the top enriched GO terms across the three pairwise comparisons.

In the A vs. B comparison (Figure 6a), DEGs were mainly enriched in the MF categories “catalytic activity” and “binding” and in the BP categories “metabolic process” and “cellular process”. In addition, CC terms such as “membrane”, “membrane part”, and “cell part” were also significantly enriched, suggesting that changes from moderate to high light intensity affected basal metabolic activity and membrane-associated cellular components.

For the A vs. C comparison (Figure 6b), in addition to persistent enrichment in “binding” and “catalytic activity”, there was strong enrichment in “cell part”, “cellular process”, and “biological regulation”, indicating that the transcriptional differences between high and low light conditions were more concentrated in cellular structure and regulatory processes. Notably, the enrichment of the “nucleic acid binding transcription factor activity” term implies that *A. kusnezoffii* may activate transcription factor-mediated regulatory mechanisms in response to high light stress.

In the B vs. C comparison (Figure 6c), the overall level of GO enrichment was relatively lower, but significant terms such as “binding”, “metabolic process”, “cellular process”, and “membrane part” were still identified, suggesting that responses under moderate-to-low light conditions were primarily associated with basic metabolic activities.

In summary, light intensity had a significant impact on the transcriptomic landscape of *A. kusnezoffii*. DEGs were predominantly enriched in GO terms related to catalytic activity, molecular binding, and metabolic regulation. The most substantial functional changes were observed under high versus low light conditions (A vs. C), providing a foundational basis for subsequent pathway enrichment and regulatory network analysis.

#### 3.3.4. KEGG Pathway Enrichment Analysis of Differentially Expressed Genes

**Figure 7:**
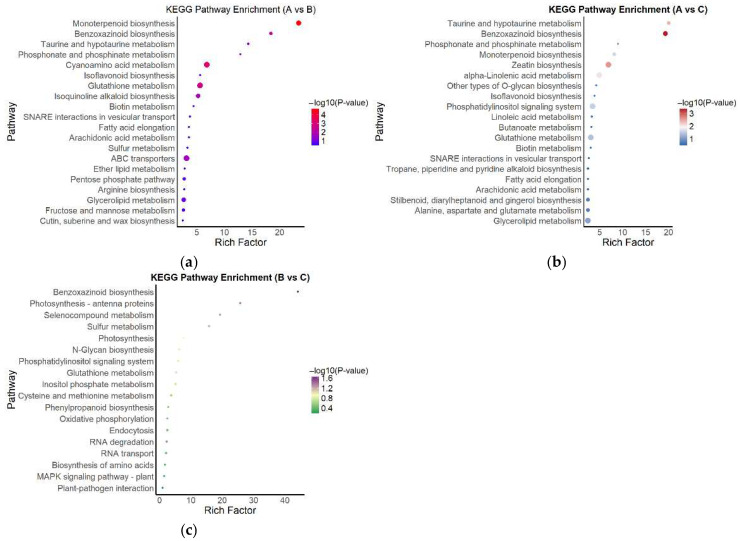
KEGG pathway enrichment analysis of DEGs in three comparison groups; (**a**) KEGG enrichment results for the A vs. B comparison group; (**b**) KEGG enrichment results for the A vs. C comparison group; (**c**) KEGG enrichment results for the B vs. C comparison group. Bubble color represents the −log_10_(*p*) value, and size indicates the number of enriched genes.

To further elucidate the potential metabolic and signaling pathways involved in the responses of *A. kusnezoffii* to different light intensities, KEGG enrichment analysis was performed on the differentially expressed genes (DEGs) identified from three pairwise comparisons: A vs. B, A vs. C, and B vs. C. The results were visualized using enrichment bubble plots (Figure 7a–c).

In the A vs. B comparison (Figure 7a), significantly enriched pathways included monoterpenoid biosynthesis, benzoxazinoid biosynthesis, taurine and hypotaurine metabolism, and phosphonate and phosphate metabolism. Several of these pathways exhibited high enrichment scores and strong statistical significance, indicating robust transcriptional changes in specific metabolic processes under high versus moderate light.

In the A vs. C comparison (Figure 7b), the enriched pathways were primarily associated with lipid metabolism, secondary metabolism, and signaling processes, including zeatin biosynthesis, linoleic acid metabolism, the phosphatidylinositol signaling system, and O-glycan biosynthesis. These results suggest that high and low light conditions induced distinct molecular responses in hormone signaling and lipid-related metabolic regulation.

In contrast, the B vs. C comparison (Figure 7c) yielded fewer enriched pathways, with significant DEGs mainly involved in photosynthesis, glutathione metabolism, the plant MAPK signaling pathway, and plant–pathogen interactions. The overall enrichment levels were relatively low, indicating a smaller transcriptomic divergence between moderate and low light conditions. The DEGs identified in this group were mostly associated with fundamental metabolic functions and stress response processes.

In summary, KEGG enrichment analysis across all three comparisons revealed that DEGs were predominantly involved in secondary metabolism, lipid biosynthesis, antioxidative responses, and signal transduction pathways. These findings imply that *A. kusnezoffii* adapts to changes in light intensity through the coordinated regulation of multiple metabolic and signaling networks.

#### 3.3.5. Trend Analysis of Differentially Expressed Genes

**Figure 8:**
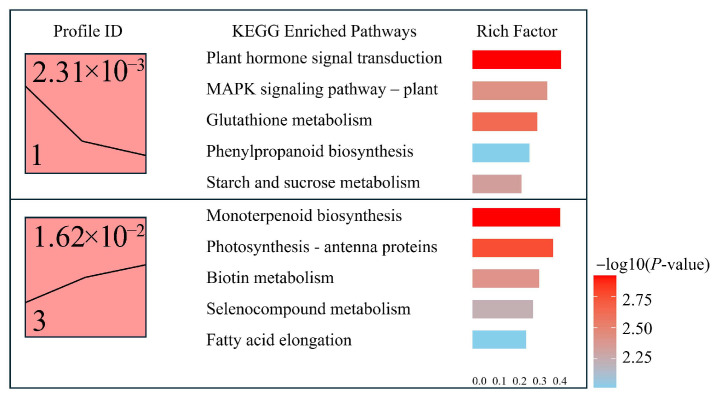
Expression trend clustering and KEGG enrichment analysis of differentially expressed genes (DEGs).

To further explore the dynamic expression patterns of differentially expressed genes (DEGs) under various light intensity treatments, trend clustering analysis was performed. Six distinct expression profiles were identified, among which Profile 1 and Profile 3 exhibited significant enrichment characteristics, with *p* values of 2.31 × 10^−3^ and 1.62 × 10^−2^, respectively (Figure 8). Genes in Profile 1 showed a continuous downregulation trend, while those in Profile 3 displayed a gradual upregulation trend, suggesting that these gene sets may play divergent biological roles across treatment stages.

KEGG enrichment analysis of genes classified in Profile 1 revealed significant enrichment in pathways such as “Plant hormone signal transduction”, “MAPK signaling pathway—plant”, “Glutathione metabolism”, “Phenylpropanoid biosynthesis”, and “Starch and sucrose metabolism”, with the highest enrichment factor reaching 0.41. These findings indicate that Profile 1 genes are predominantly involved in hormone signaling and stress-related metabolic processes, potentially contributing to environmental response and metabolic reprogramming.

In contrast, Profile 3 genes were mainly enriched in pathways including “Monoterpenoid biosynthesis”, “Photosynthesis—antenna proteins”, “Biotin metabolism”, “Selenocompound metabolism”, and “Fatty acid elongation.” These results suggest that the upregulated genes in Profile 3 may be associated with monoterpenoid biosynthesis, the regulation of photosynthetic machinery, and fatty acid metabolism. These processes likely contribute to physiological recovery and the maintenance of cellular homeostasis during light stress adaptation.

#### 3.3.6. Expression Patterns of Genes Involved in Light Signaling During Chloroplast Development in *A. kusnezoffii*

To investigate the regulatory mechanisms of light signaling pathways during chloroplast development in *A. kusnezoffii*, a total of 36 key regulatory genes with significant differential expressions were identified from the transcriptome dataset. These genes included light receptors (*PHY* and *Cry*), negative regulators (*COP1* and *SPA1*), transcriptional regulators (*PIFs* and *HY5*), and downstream genes involved in chloroplast biogenesis and division (Cp- and PhANGs). The expression levels of these light-responsive components varied significantly under different light intensities, showing distinct expression patterns.

In the A vs. B comparison (high vs. moderate light), 11 *PHY* genes were upregulated and 5 were downregulated. Among the *COPI* genes, 8 were upregulated and 3 were downregulated. For the *PIFs* family, 4 genes showed upregulation while 2 were downregulated. Additionally, one *HY5* gene was upregulated, and one was downregulated. These findings suggest that moderate light conditions, compared to high light, may enhance the perception and transmission of light signals by activating light receptors and negative regulators.

In the A vs. C comparison (high vs. low light), *PHY* gene expression was more balanced, with 8 genes upregulated and 8 downregulated. In the *COPI* family, 7 genes were upregulated and 4 downregulated; *PIFs* maintained a similar pattern with 4 upregulated and 2 downregulated genes. Notably, both *HY5* genes were downregulated under low light conditions, indicating that suppression of *HY5* may affect the transcriptional regulation of chloroplast-associated genes during light stress.

**Figure 9:**
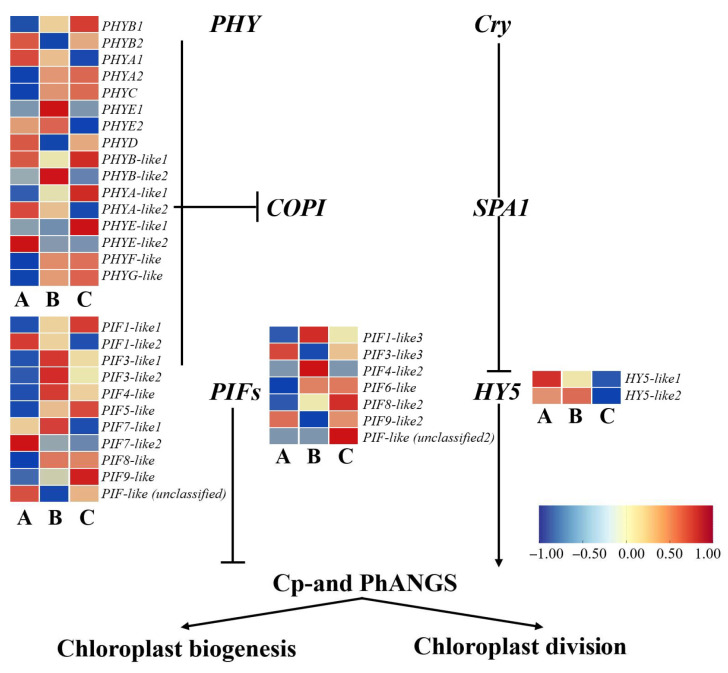
Schematic model of light signaling-regulated chloroplast development in *A. kusnezoffii* under different light intensities. Differentially expressed genes (DEGs) involved in photoreceptors (*PHY* and *Cry*), transcription factors (*PIFs* and *HY5*), and key signaling components (*COP1* and *SPA1*) affect the transcription of chloroplast-related genes (Cp-and PhANGs), regulating chloroplast biogenesis and division. Heatmaps show gene expression changes in samples A (low light), B (moderate light), and C (high light). Color bar indicates log_2_ fold change (scale: −1 to 1).

In the B vs. C comparison (moderate vs. low light), 11 *PHY* genes were upregulated while 4 were downregulated. For *COPI*, 4 genes were upregulated and 6 downregulated. Among the *PIFs*, 4 genes were upregulated and 2 downregulated, while 2 *HY5* genes were upregulated. These results suggest that *PHY* genes are generally more active under moderate light, whereas *COPI* expression tends to be suppressed under low light conditions (Figure 9).

Overall, the expression of positive regulators, such as *PHY* and *HY5*, was elevated under moderate light, while negative components, like *COPI* and *PIFs*, showed limited upregulation or moderate downregulation. This balance likely promotes the expression of chloroplast biogenesis and function-related genes (Cp- and PhANGs). In contrast, strong downregulation of *HY5* under low light may hinder the normal progression of photomorphogenesis, thereby affecting chloroplast division and development.

#### 3.3.7. Expression Patterns of Photosynthesis-Related Genes Regulated by Light Signaling in *A. kusnezoffii*

To elucidate the molecular mechanisms by which light signaling regulates photosynthetic processes in *A. kusnezoffii*, a total of 34 differentially expressed genes (DEGs) involved in light reactions, dark reactions, and the carotenoid cycle were identified and analyzed based on clustering and functional annotation.

In the A vs. B comparison (high light vs. moderate light), 15 DEGs related to the light reactions were identified. Notably, *Lhcb1* and *PsbS1* were downregulated, while *Lhcb4* and *Psb28* were upregulated, suggesting that moderate light enhances the expression of certain light-harvesting antenna and reaction center proteins to improve energy capture. Significant changes were also observed in photosystem II components, including *PsbC* (three upregulated and one downregulated), *PsbQ* (one upregulated and three downregulated), and *PsbR* (two downregulated), indicating a differential regulation of PSII subunits under varying light intensities. In the dark reaction phase, both *Lhca1* and *Lhca5* were downregulated. Subunits of the ATP synthase complex, such as *atpC* (three upregulated and two downregulated), *atpD* (two downregulated), *atpF2* (one downregulated), and *PetC* (two downregulated), also showed notable changes. In contrast, *petD* and *petJ* were upregulated, indicating that genes involved in electron transport and energy conversion were partially activated under moderate light conditions.

A similar trend was observed in the A vs. C comparison (high light vs. low light). *Lhcb1* was downregulated, while *Lhcb4* and *Lhcb5* were upregulated. *Psb28* was upregulated, *PsbS1* was downregulated, and *PsbC* maintained the same trend (three upregulated and one downregulated). Both *PsbQ* and *PsbR* exhibited two upregulated and two downregulated genes, highlighting their dynamic regulation under different light regimes. In the dark reaction phase, *Lhca1* was upregulated while *Lhca5* was downregulated. Other genes included *atpC* (two upregulated and three downregulated), *atpF2* (one downregulated), *atpD* (two downregulated), *PetC* (two downregulated), *petD* (one upregulated and one downregulated), *petJ* (upregulated), and *PsaN* and *PsaF* (both upregulated), suggesting that partial activation of the energy transformation machinery may occur under low light. Interestingly, three *PHOT2* (blue-light receptor) genes were upregulated, and one was downregulated, implying enhanced blue-light signaling under shade conditions.

**Figure 10:**
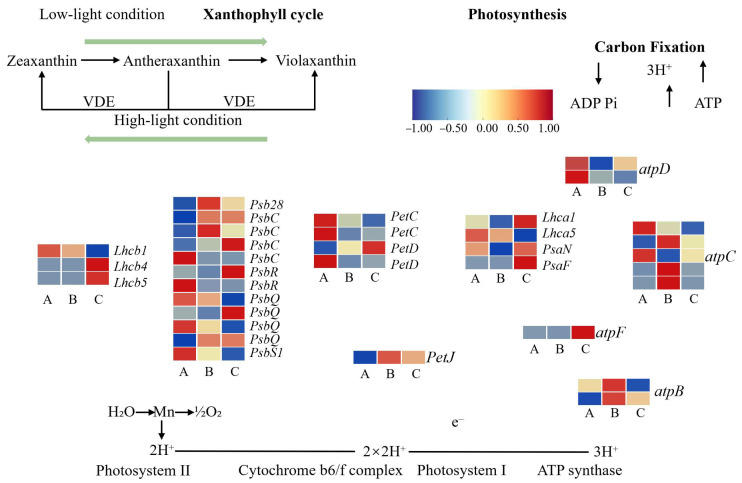
Transcriptomic regulation of photosynthesis-related genes in *A. kusnezoffii* under different light intensities. Differentially expressed genes (DEGs) involved in the xanthophyll cycle, the light harvesting complex (LHC), photosystems I and II, the cytochrome b6/f complex, ATP synthase, and carbon fixation were identified and mapped. Heatmaps indicate expression patterns under low (A), moderate (B), and high (C) light conditions. Color scale represents log_2_ fold change (from −1 to 1).

In the B vs. C comparison (moderate light vs. low light), similar expression patterns were observed. *Lhcb1* was downregulated, *Lhcb4* and *Lhcb5* were upregulated, *PsbS1* was downregulated, and *Psb28* was upregulated. The expression of *PsbC*, *PsbQ*, and *PsbR* showed a mixture of up- and downregulated patterns. In the dark reaction pathway, *Lhca1* was upregulated and *Lhca5* downregulated; *atpC* (two upregulated and three downregulated), *atpD* (one upregulated and one downregulated), *atpF2* (one upregulated), *atpB* (two downregulated), *PetC*, *petD*, *PsaN*, and *PsaF* were all upregulated, while *petJ* was downregulated (Figure 10).

In summary, under moderate light intensity (620 lx), several key genes involved in both light and dark reactions—including *Psb* family members, *Pet* genes, ATP synthase subunits, and photosystem I/II proteins—were more actively expressed. This expression profile may underlie the enhanced photosynthetic efficiency observed under optimal light conditions. In contrast, both high (790 lx) and low (450 lx) light intensities appeared to suppress the expression of photosynthesis-related genes, potentially impairing chloroplast function and energy acquisition.

#### 3.3.8. Expression Patterns of Genes Involved in Photomorphogenesis of *A. kusnezoffii* Under Different Light Intensities

To gain deeper insights into the molecular regulatory mechanisms underlying the photomorphogenesis of *A. kusnezoffii* under varying light intensities, we constructed an integrated regulatory model based on transcriptome data, incorporating both light signal transduction and hormone biosynthesis pathways. A total of 57 differentially expressed genes (DEGs) were identified, involving multiple levels, including photoreceptors, transcription factors, and hormone biosynthetic pathways.

In the A vs. B comparison (high vs. moderate light), *PHYB* showed the most pronounced expression changes among photoreceptors, with four genes upregulated and six downregulated. One *PHYC* gene was downregulated, while *PHYE* genes showed bidirectional regulation (two upregulated and two downregulated). Similarly, *PHOT2* displayed two upregulated and two downregulated genes, indicating a complex and dual-mode regulatory response to moderate light conditions. Among downstream transcription factors, one *PIF3* gene was downregulated, while *PIF1* genes showed four upregulated and one downregulated, suggesting the central role of *PIFs* in modulating photomorphogenesis under moderate light.

An expression analysis of hormone biosynthesis-related genes revealed three upregulated and seven downregulated genes in the jasmonic acid (JA) pathway, seven upregulated and four downregulated genes in the auxin (IAA) pathway, and four downregulated genes in the gibberellin (GA) pathway. In the brassinosteroid (BR) pathway, two genes were upregulated and two downregulated. These results suggest that moderate light may promote favorable morphological adaptations in *A. kusnezoffii* through a coordinated regulation of JA, IAA, GA, and BR biosynthesis.

In the A vs. C comparison (high vs. low light), *PHYB* genes included three upregulated and six downregulated members; one *PHYC* gene was downregulated, and *PHYE* genes exhibited expression patterns of three upregulated members and one downregulated. Regarding the transcription factors, *PIF3* was downregulated, *PIF7* was upregulated, and *PIF1* genes included three upregulated and two downregulated members. In hormone-related pathways, one gene involved in JA biosynthesis was upregulated and nine downregulated; IAA biosynthesis included nine upregulated and two downregulated genes; all four GA-related genes were downregulated; and BR biosynthesis showed two upregulated and two downregulated genes. These results suggest that under low light, *A. kusnezoffii* may suppress JA and GA biosynthesis while enhancing IAA signaling to adapt its developmental strategy to shade conditions.

**Figure 11:**
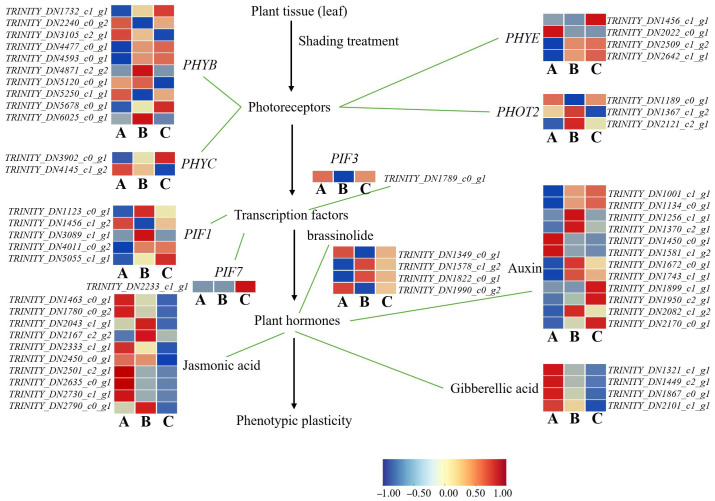
Transcriptomic analysis of light-regulated photomorphogenesis-related genes in *A. kusnezoffii*. The proposed regulatory network shows the expression patterns of the photoreceptors (e.g., *PHYB*, *PHYC*, *PHYE*, and *PHOT2*), transcription factors (e.g., *PIF1*, *PIF3*, and *PIF7*), and hormone biosynthesis pathways (auxin, gibberellic acid, jasmonic acid, and brassinolide) involved in phenotypic plasticity in response to different light conditions. Heatmaps illustrate gene expression levels under low (A), moderate (B), and high (C) light intensities, with the color scale representing log_2_ fold change (−1 to 1).

In the B vs. C comparison (moderate vs. low light), photoreceptor genes remained highly responsive: six *PHYB* genes were upregulated and four downregulated; *PHYC* was downregulated; *PHYE* showed three upregulated and one downregulated gene; and *PHOT2* had three upregulated and one downregulated gene. For transcription factors, both *PIF3* and *PIF7* were upregulated by one gene each, while *PIF1* genes showed three upregulated and two downregulated members. In terms of hormone-related DEGs, the JA pathway included three upregulated and seven downregulated genes; the IAA pathway had five upregulated and seven downregulated genes; all four GA biosynthetic genes were downregulated; and BR biosynthesis showed two upregulated and two downregulated genes (Figure 11).

Taken together, these results suggest that *A. kusnezoffii* modulates photomorphogenesis under different light environments by regulating the expression of key photoreceptors (*PHYB*, *PHYE*, and *PHOT2*), downstream transcription factors (*PIF1*, *PIF3*, and *PIF7*), and genes involved in multiple hormone biosynthesis pathways. This coordinated transcriptional reprogramming enables the plant to perceive and integrate light signals, thereby regulating plasticity in leaf development. The findings provide important molecular insights into the adaptive responses of *A. kusnezoffii* to light intensity variation.

### 3.4. Relative Expression Analysis of Key Genes

To validate the accuracy of transcriptome sequencing results from *A. kusnezoffii* leaves, ten differentially expressed genes (DEGs) were randomly selected for quantitative real-time PCR (qRT-PCR) analysis. Specific primers were designed for each target gene. Among them, *GA2ox* and *LHCB1* were upregulated genes, while *PFK*, *NCED*, *PP2C*, *DELLA*, *AMY*, *LHCB17*, *ATPβ*, and *PetA* were downregulated genes.

**Figure 12:**
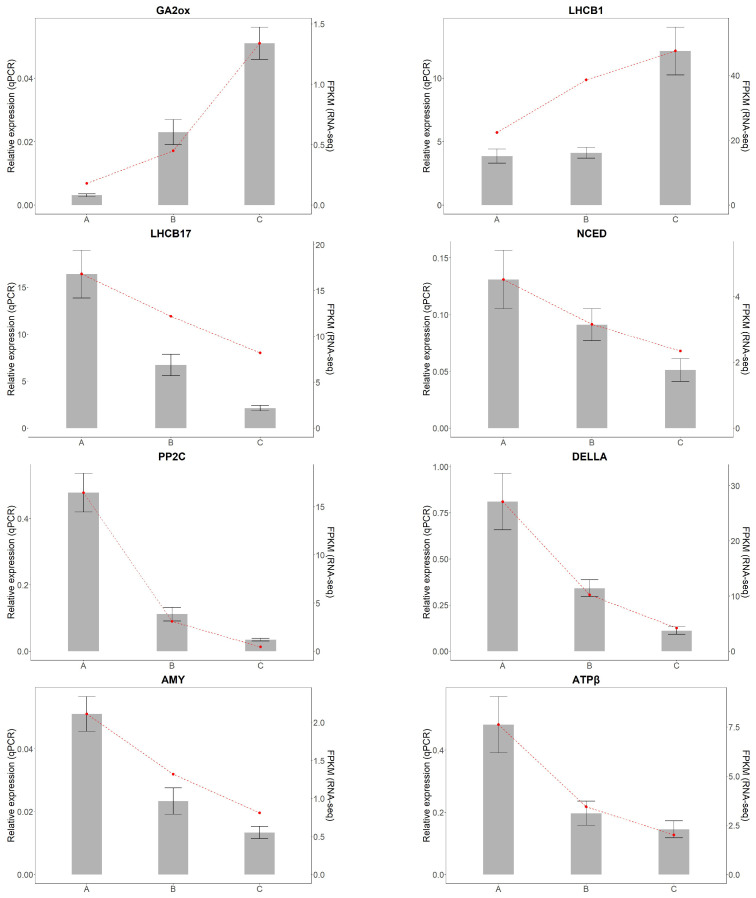

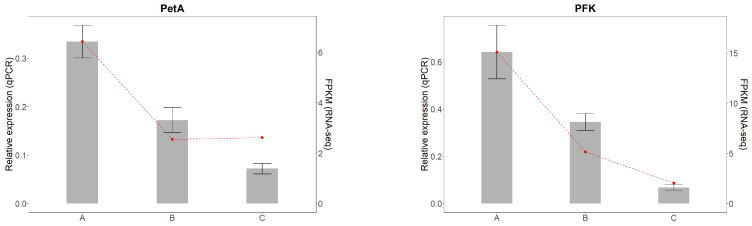
Validation of differentially expressed genes by qRT-PCR.Gray bars represent relative expression levels determined by qRT-PCR (left y-axis), and the red dashed line represents corresponding FPKM values from RNA-seq (right y-axis). Error bars indicate standard deviation (SD) of three biological replicates.

The qRT-PCR results show that the relative expression patterns of all ten genes were consistent with the RNA-Seq data, confirming the reliability of the transcriptomic analysis (Figure 12).

## 4. Discussion

### 4.1. Regulation of Leaf Anatomical Structure in A. kusnezoffii Under Different Light Intensities

Light is one of the most critical environmental factors influencing the formation and functional differentiation of plant leaf structures. Plants often adapt to variations in light availability through plastic adjustments in leaf morphology and tissue organization. In the present study, *A. kusnezoffii* grown under moderate light intensity (620 lx) exhibited a significantly greater upper and lower epidermal thickness, palisade tissue thickness, and palisade-to-spongy tissue ratio than those grown under high (790 lx) or low (450 lx) light conditions. These anatomical features suggest a more coordinated and optimized leaf structure under moderate light, consistent with previous findings in other plant species showing enhanced photosynthetic architecture under optimal irradiance.

For instance, in *Tradescantia pallida* cv. *purpurea*, decreasing light intensity significantly reduced leaf lamina and mesophyll thickness, particularly under 40 μmol·m^−2^·s^−1^, where palisade and spongy tissue layers were less developed, indicating that low light impairs the development of photosynthetic tissues [14]. Similarly, in *Camellia oleifera*, moderate light treatment led to the optimal leaf thickness, specific leaf area (SLA), and compactness of palisade cells, supporting moderate light as the most favorable irradiance for this species [15]. These observations align with our results, where the palisade tissue thickness under 620 lx was significantly greater than in other treatments, suggesting that moderate light enhances the development of photosynthetic structures in *A. kusnezoffii*.

In terms of epidermal structure, plant species display variable responses to light intensity. In *Nicotiana tabacum*, high light increased the upper epidermal thickness by 19.7% without affecting the lower epidermis, indicating differential responsiveness across epidermal layers [16]. In contrast, in *A. kusnezoffii*, both the upper and lower epidermal layers were significantly thickened under 620 lx, suggesting that moderate light promotes the development of protective tissue in a more balanced manner.

Furthermore, the highest total leaf thickness was observed under 790 lx, consistent with studies on *N*. *tabacum* [17] and *Rhododendron* spp. [18], where high light generally promoted leaf thickening. In *N. tabacum*, high light increased the leaf thickness by 32.1%, with palisade and spongy tissues increasing by 49.0% and 29.8%, respectively, demonstrating the sensitivity of mesophyll structure to light variation. However, this thickening often results from cell elongation rather than division and may not enhance photosynthetic efficiency but instead may represent a passive adaptation to photooxidative stress.

The palisade-to-spongy tissue thickness ratio is an important anatomical index reflecting the balance between photosynthetic and gas exchange tissues. In this study, the 620 lx treatment yielded the highest ratio, indicating an optimized distribution of mesophyll components to support photosynthetic efficiency. Previous studies have shown that under efficient photosynthetic conditions, plants tend to increase palisade tissue thickness and cell density to enhance light capture and carbon fixation [19].

In conclusion, *A. kusnezoffii* exhibited more favorable leaf anatomical traits under moderate light intensity (620 lx), reflecting enhanced adaptability to optimal irradiance. This adjustment may involve increased epidermal thickness for protection, enhanced palisade development for efficient photosynthesis, and the maintenance of spongy tissue structure for gas exchange. These findings provide theoretical support for understanding light adaptation in *A. kusnezoffii* and optimizing light management in artificial cultivation.

### 4.2. Physiological Response Mechanisms of A. kusnezoffii Under Different Light Intensities

This study systematically revealed the physiological adaptation mechanisms of *A. kusnezoffii* to varying light intensities (450, 620, and 790 lx) by analyzing malondialdehyde (MDA) content, antioxidant enzyme activity, and the accumulation of osmoregulatory substances. Overall, *A. kusnezoffii* exhibited the most favorable oxidative defense and metabolic activity under moderate light intensity (620 lx), whereas low light conditions (450 lx) led to intensified oxidative stress, suppression of protective systems, and disturbances in carbon metabolism.

In terms of membrane lipid peroxidation, the MDA content in the leaves significantly increased under 450 lx, suggesting that low light induced oxidative damage to the membrane system. Similar findings were reported by Dong et al., who observed a marked increase in MDA levels in *Triticum aestivum* grown under low light conditions during grain filling, ultimately affecting yield formation [20]. Likewise, Omer et al. demonstrated that low light resulted in elevated MDA content in *Origanum onites*, accompanied by a reduced photosynthetic rate (Pn) and cellular ultrastructural abnormalities, highlighting the persistent stress imposed on membrane systems by insufficient irradiance [21].

Regarding the antioxidant defense system, the activities of CAT, POD, and SOD were highest under 620 lx and significantly suppressed under 450 lx, indicating that moderate light effectively activates the reactive oxygen species (ROS)-scavenging system. Chen et al. reported that in lettuce, POD activity increased rapidly under early-stage low light to mitigate ROS accumulation but decreased in prolonged low light conditions, leading to MDA accumulation [22]. In this study, *A. kusnezoffii* under 450 lx similarly exhibited reduced CAT and POD activities and elevated MDA levels, consistent with this dynamic pattern. Johkan et al. found that in *Lactuca sativa*, CAT and POD activities increased in response to enhanced light intensity, underscoring the light-sensitive and phased regulation of protective enzymes [23].

In addition to antioxidant enzymes, *A. kusnezoffii* accumulated the highest levels of soluble protein, soluble sugars, and starch under 620 lx, indicating that moderate light promoted photosynthetic product synthesis and enhanced the osmotic adjustment capacity. Chen et al. also demonstrated that high light intensity (200 μmol·m^−2^·s^−1^), in combination with optimal temperature conditions, significantly increased carbohydrate accumulation and nutritional quality in lettuce [22]. Similarly, Albayrak and Çamaş identified that under 420–430 kcal/cm^2^ and 20 °C, *Beta vulgaris* achieved the highest dry matter accumulation, emphasizing the synergistic effect of moderate light and warm temperatures on carbon metabolism efficiency. In line with this, *A. kusnezoffii* under 620 lx exhibited significantly higher levels of soluble sugars (66.0 mg/g) and starch (21.8 mg/g), contributing to enhanced osmotic regulation and energy storage.

Interestingly, proline content did not differ significantly across the treatments, which is consistent with findings on *T*. *aestivum*, where proline accumulation occurred rapidly in response to drastic light fluctuations but remained stable under constant low or high light [20]. This suggests that in *A. kusnezoffii*, proline may primarily function as a short-term stress-responsive osmolyte rather than a long-term regulatory metabolite.

A comparative analysis with other crops indicated that *A. kusnezoffii* displays a “moderate light adaptation” profile. Under 620 lx, enzymatic activity, metabolic product accumulation, and membrane stability were all optimized. This pattern parallels results on *B*. *vulgaris*, which exhibited optimal photosynthetic performance and structural features under 30–50% full light [24]. Similarly, studies on *Coriandrum sativum* and *Solanum lycopersicum* by Wang et al. and Fan et al., respectively, reported that under moderate light intensity (200–300 μmol·m^−2^·s^−1^), leaf anatomical development and Pn were enhanced along with antioxidant capacity [25,26]—findings consistent with our observations of *A. kusnezoffii* under 620 lx.

In contrast, the 450 lx treatment led to reduced physiological activity and stress-related impairments, as also observed in *C*. *sativum*, where low light caused early bolting and a decline in vitality [25]. Similarly, both Johkan et al. [23] and Albayrak et al. [24] reported that low light limited the excitation efficiency of Photosystem II and the capacity for carbon assimilation.

In summary, *A. kusnezoffii* exhibits a clear physiological threshold in response to light intensity, with 620 lx representing the optimal level under current experimental conditions. This light level significantly enhances antioxidant capacity, carbon metabolism, and cellular homeostasis, while 450 lx induces oxidative stress and restricts metabolic performance. Integrating findings across species, we propose that *A. kusnezoffii* is best suited for cultivation under moderate and stable irradiance environments. Alternatively, controlled-environment agriculture with moderate-intensity LED lighting (approximately 200–300 μmol·m^−2^·s^−1^) may provide optimal conditions for achieving both high productivity and resource efficiency.

### 4.3. Multi-Level Transcriptomic Response Mechanisms of A. kusnezoffii Under Different Light Intensities

Plants modulate their growth, development, and metabolic status through complex mechanisms of light perception and signal integration to adapt to variable light environments. In this study, *A. kusnezoffii* exhibited a typical light-responsive regulatory pattern under different light intensities (790 lx, 620 lx, and 450 lx), encompassing multiple layers, such as light signal perception, photosystem reconfiguration, hormone signaling crosstalk, and metabolic pathway remodeling. These findings highlight the species’ high sensitivity and multi-layered adaptive regulation in response to environmental light fluctuations.

Under moderate light treatment (620 lx), positive regulators, such as *PHY* and *HY5*, showed enhanced expression, whereas negative regulators, such as *COP1* and *PIFs*, were suppressed. This suggests a classical light signaling axis characterized by “photoreceptor activation—repression of negative regulators—accumulation of transcription factors.” This regulatory framework aligns with findings on *Epimedium pseudowushanense*, where *CHS* and *F3’H* genes modulate flavonoid biosynthesis under different light intensities [27], and is also consistent with the activation of *PAL* and phenylpropanoid pathway genes under moderate light in *Marsdenia tenacissima*, promoting chlorogenic acid accumulation [28].

The transcriptional responses of *A. kusnezoffii* extended beyond light perception and prominently affected the photosynthetic system. Under moderate and low light conditions, multiple genes encoding Photosystem II antenna proteins and reaction center subunits (e.g., *Lhcb4*, *PsbC*, and *Psb28*) were upregulated, suggesting an adaptive enhancement in light-harvesting capacity to maintain photosynthetic equilibrium. Similar regulatory mechanisms have been reported in shade-tolerant species, such as *T*. *pallida* [29] and *Camellia sinensis* [30]. Conversely, key components of the ATP synthase complex and electron transport chain showed downregulated expressions, implying that *A. kusnezoffii* may actively suppress excessive energy synthesis to avoid oxidative damage—a strategy previously observed in *T*. *aestivum* [31] and *Taxus chinensis* [32] under high light or complex LED light spectra.

In addition to structural modulation, *A. kusnezoffii* displayed a distinct pattern of signal integration. Expression patterns of *PIF1/3/7* were tightly linked to hormone signaling pathways, especially under low light conditions, where genes involved in auxin (IAA) biosynthesis were upregulated, whereas gibberellin (GA)-related genes were suppressed. This forms a characteristic “high IAA–low GA” hormone response profile, which has been similarly reported under blue light for *Brassica rapa* var. *purpurea* [33], in young leaves of *C. sinensis* under low light [34], and in *Angelica dahurica* under shaded conditions [35]. The interaction among light, hormones, and morphology constitutes a core molecular framework for photomorphogenesis.

At the metabolic level, KEGG enrichment analysis showed that DEGs in *A. kusnezoffii* were significantly enriched in pathways such as monoterpenoid biosynthesis, glutathione metabolism, and phosphatidylinositol signaling, indicating that the species may fine-tune its antioxidant capacity and membrane lipid composition to cope with light stress. These findings are in agreement with prior studies on light-regulated secondary metabolism in *L*. *sativa* [36] and *Zea mays* [37]. Additionally, carotenoid-related pathways in *A. kusnezoffii* exhibited expression patterns comparable to those observed during developmental transitions in *N*. *tabacum* [38], suggesting a possible enhancement in protective pigments or barrier functions under variable light conditions.

A trend clustering analysis further revealed stage-specific light responses in *A. kusnezoffii*. Genes in Profile 1, including those enriched in “MAPK signaling”, “glutathione metabolism”, and hormone-related pathways, were continuously downregulated in early stress phases, possibly indicating a shift toward steady-state regulation following stress perception. In contrast, Profile 3 showed sustained upregulation of genes associated with “photosynthesis” and “fatty acid metabolism”, suggesting that restorative metabolic processes were activated during the mid-to-late phases. This “early suppression–later reconstruction” model is highly consistent with transcriptomic module switching patterns observed in *T. aestivum* [31] and *Z. mays* [37] under diverse light qualities and time courses, emphasizing the time-dependent and modular nature of light-responsive regulation in plants.

In summary, *A. kusnezoffii* demonstrates a multi-dimensional regulatory strategy under different light intensities, centered on light signal transduction, bridged by hormone integration, and manifested through metabolic reprogramming. This integrative plasticity constitutes an effective adaptation mechanism for survival and growth in the variable light environments typical of alpine habitats.

## 5. Conclusions

In this study, we demonstrated that *A. kusnezoffii* exhibits a coordinated physiological and transcriptomic response to varying light intensities, with moderate light (620 lx) inducing the most favorable effects. Moderate light promoted an optimized anatomical structure, improved antioxidant defense, and enhanced the accumulation of key osmolytes and photosynthetic products. Transcriptome analysis revealed that DEGs were significantly enriched in pathways related to photosynthesis, hormone signaling, and stress responses, with key photoreceptors (*PHY* and *PHOT2*), transcription factors (*HY5* and *PIFs*), and hormone biosynthesis genes showing light-dependent regulation. The species displayed a modular and time-resolved gene expression pattern, supporting its adaptability to fluctuating light environments. These results suggest that *A. kusnezoffii* is best suited to moderate and stable light conditions and that artificial cultivation strategies should prioritize controlled lighting regimes (e.g., 200–300 μmol·m^−2^·s^−1^) to maximize growth and medicinal quality.

## Figures and Tables

**Table 1:** Primer sequences for qRT-PCR validation of transcriptome data.

Gene	Forward Primer	Reverse Primer
*PFK*	GATTTGCTTGCCATTCTTCTCTC	CTTGCAGTGAACCCACTC
*NCED*	TGTTCCCGAATGTCCGTGT	TTCGCTCCGTTACGAAGA
*PP2C*	TGGTGTTCGGTGGGAAAGAA	GACTTTGCCACCGGATA
*GA2ox*	CTCCTGACCCTACTGACTT	GGGCCTTGTTGAGTTTGAT
*DELLA*	TCCTCTGACATGGTTCACTAC	AGTGTCGAATCGGAGGAGC
*AMY*	AGACTTGAGAGAGGAGAGGAA	ACAATAGGTGCTGGAGTCTT
*LHCB17*	ATGGGGGCTCTCTTCTTCTCTCT	TTAGTGAGCACGGTGTCC
*LHCB1*	ATGGCCCTCTTGATGTTTGC	TTAGTGAGCACGGTGTCC
*ATPβ*	ATGGCGAGACCTTGGTGTTGT	TTAGTGGTCTCGGAAGTTTG
*PetA*	ATGGAAGGTTCCATCCTCCA	TCAGTGAGCACGGTGTCC

**Table 2:** Statistical results of *A. kusenezoffii* leaf sequencing.

Sample	Clean Reads	Clean Bases	GC Content	%≥30
A1	20,913,066	6,253,938,597	46.04%	95.06%
A2	20,937,218	6,271,699,310	45.75%	95.17%
A3	21,340,129	6,391,249,626	45.53%	95.10%
B1	20,952,999	6,277,146,897	45.67%	94.81%
B2	20,283,257	6,077,272,468	45.67%	95.49%
B3	21,234,781	6,359,402,361	45.79%	94.81%
C1	21,344,169	6,389,542,876	46.00%	94.37%
C2	20,223,069	6,055,680,316	45.82%	95.49%
C3	22,691,284	6,795,199,966	45.65%	95.35%

## Data Availability

Due to the confidentiality of the project, relevant data cannot be made publicly available at this time. The data will be shared in accordance with the relevant regulations at an appropriate time.
